# Somatic comorbidity and physical frailty in elderly with medically unexplained symptoms

**DOI:** 10.1192/j.eurpsy.2022.1214

**Published:** 2022-09-01

**Authors:** M. Arts

**Affiliations:** GGZWNB, Psychiatry, Bergen op Zoom, Netherlands

**Keywords:** Frailty, Somatic, MUS, comorbidity

## Abstract

**Introduction:**

Reported prevalence rates of medically unexplained symptoms (MUS) in people aged ≥65 years range between 1.5 and 18%. People with MUS often describe a low quality of life and frequently suffer from co-morbid anxiety and depressive disorders. In our pilot study on older patients with MUS, the level of somatic comorbidity as well as frailty parameters were significantly higher among patients with MUS which was partially explained by a somatic origin compared to patients with MUS for which no explanation at all was found.

**Objectives:**

The objective of this study was to examine the level of frailty and somatic comorbidity in older patients with medically unexplained symptoms (MUS) and compare this to patients with medically explained symptoms (MES).

**Methods:**

Frailty was assessed according to Fried’s criteria (gait speed, handgrip strength, unintentional weight loss, exhaustion, and low physical activity), somatic comorbidity according to the self-report Charlson Comorbidity Index and the number of prescribed medications.

**Results:**

Although MUS-patients had less physical comorbidity compared to MES-patients, they were prescribed the same number of medications. Moreover, MUS-patients were more often frail compared to MES-patients. Among MUS-patients, physical frailty was associated with the severity of unexplained symptoms, the level of hypochondriacal beliefs, and the level of somatisation.

**Conclusions:**

Despite a lower prevalence of overt somatic diseases, MUS-patients are more frail compared to older MES-patients. These results suggest that at least in some patients age-related phenomena might be erroneously classified as MUS, which may affect treatment strategy.

**Disclosure:**

No significant relationships.

